# Structural Analysis of the Hg(II)-Regulatory Protein *Tn501* MerR from *Pseudomonas aeruginosa*

**DOI:** 10.1038/srep33391

**Published:** 2016-09-19

**Authors:** Dan Wang, Shanqing Huang, Pingying Liu, Xichun Liu, Yafeng He, Weizhong Chen, Qingyuan Hu, Tianbiao Wei, Jianhua Gan, Jing Ma, Hao Chen

**Affiliations:** 1 Coordination Chemistry Institute and the State Key Laboratory of Coordination Chemistry, School of Chemistry and Chemical Engineering, Collaborative Innovation Center of Chemistry for Life Sciences, Nanjing University, Nanjing 210093, P. R. China; 2 Institute of Theoretical and Computational Chemistry, School of Chemistry and Chemical Engineering, Nanjing University, Nanjing 210093, P. R. China; 3 Department of Chemical Physics, University of Science and Technology of China, Hefei 230026, P. R. China; 4 School of Life Sciences, Fudan University, Shanghai 200433, P. R. China

## Abstract

The metalloprotein MerR is a mercury(II)-dependent transcriptional repressor-activator that responds to mercury(II) with extraordinary sensitivity and selectivity. It’s widely distributed in both Gram-negative and Gram-positive bacteria but with barely detectable sequence identities between the two sources. To provide structural basis for the considerable biochemical and biophysical experiments previously performed on *Tn501* and *Tn21* MerR from Gram-negative bacteria, we analyzed the crystal structure of mercury(II)*-*bound *Tn501* MerR. The structure in the metal-binding domain provides *Tn501* MerR with a high affinity for mercury(II) and the ability to distinguish mercury(II) from other metals with its unique planar trigonal coordination geometry, which is adopted by both Gram-negative and Gram-positive bacteria. The mercury(II) coordination state in the C-terminal metal-binding domain is transmitted through the allosteric network across the dimer interface to the N-terminal DNA-binding domain. Together with the previous mutagenesis analyses, the present data indicate that the residues in the allosteric pathway have a central role in maintaining the functions of *Tn501* MerR. In addition, the complex structure exhibits significant differences in tertiary and quaternary structural arrangements compared to those of *Bacillus* MerR from Gram-positive bacteria, which probably enable them to function with specific promoter DNA with different spacers between −35 and −10 elements.

The heavy metal mercury easily accumulates in living organisms and causes substantial toxicity because of its high affinity for the functional thiolate-groups of many enzymes^1^. When exposed to an overdose of mercury, certain bacteria use specific resistance systems to promote survival^2^. The best-studied mercury resistance *mer* operons were first characterized in the transposons *Tn501* and *Tn21* from the Gram-negative bacterium *Pseudomonas aeruginosa* and the *Shigella flexneri* R100 plasmid, respectively^3^,^4^. The *mer* operons comprise a suite of structural genes involved in transporting and transforming inorganic and organic mercury^5^,^6^,^7^. In Gram-negative bacteria, the typical structural genes *merTP*(*C*)*AD* encode mercury(II) transporters MerT, MerP, and MerC, the mercury(II) reductase enzyme MerA, and a probable repressor MerD, which confer narrow-spectrum mercury resistance. This narrow-spectrum mercury resistance involves the reduction of mercury(II) and resistance to several organomercurials including merbromin and fluorescein mercuric acetate^6^,^8^. The *mer* operons in certain bacteria also contain the structural gene *merB*, which encodes the organomercurial lyase responsible for biotransformation of phenylmercuric acetate and methyl mercury, thus conferring broad-spectrum mercury resistance^8^.

The *mer* operons are under the regulation of the mercury(II)-dependent transcriptional repressor-activator MerR. Regardless of whether mercury(II) is present, the MerR protein binds a palindromic DNA sequence at the −35 and −10 regions of its promoter DNA^9^,^10^. The spacer between the −35 and −10 elements is longer than the optimal 17 ± 1 bp for recognition by σ^70^ RNA polymerase^11^. Thus, in the absence of mercury(II), MerR still represses the transcription of the structural genes by capturing RNA polymerase in a closed inactive state^12^,^13^. Upon mercury(II) binding, the conformation changes of MerR induce a local underwinding of the promoter DNA, which facilitates the formation of an open complex by RNA polymerase to initiate transcription^10^. The regulator MerR also regulates its own transcription from the *merR* promoter, regardless of whether mercury(II) is present^12^,^14^. MerR is a representative metalloregulatory protein that exhibits high sensitivity and selectivity to mercury(II) *in vivo* and *in vitro*. It recognizes submicromolar mercury(II), even in the presence of millimolar concentrations of small molecular thiol-competing ligands, and shows hundreds of times more selectivity towards mercury(II) than other metal ions^5^,^10^,^15^. Thus, understanding the structural rationale underlying this property and determining how it functions is of interest.

Since the identification of the MerR protein 30 years ago, several studies have been conducted to obtain structural information for this protein^11^. Biochemical and biophysical experiments performed with *Tn501* and *Tn21* MerR have indirectly revealed some of structural features^13^,^16^,^17^,^18^,^19^,^20^,^21^,^22^,^23^,^24^,^25^,^26^,^27^. The three-dimensional structure of MerR was not determined until a breakthrough was made in a structural study of *Bacillus* MerR^28^. However, the MerR proteins from Gram-positive and Gram-negative sources have barely detectable sequence identities (Supplement Fig. S1)^11^. The crystal structure of *Bacillus* MerR from Gram-positive bacteria can’t provide structural rationalizations for the large numbers of previous mutagenesis analyses performed in Gram-negative bacteria (Supplement Fig. S1). Moreover, the MerR proteins from two different sources may adapt different spatial structures to function as the repressor and mercury(II)-responsive transcription activator. Therefore, determining the three-dimensional structure of MerR from Gram-negative bacteria has profound significance. The *Tn501* and *Tn21* MerR from Gram-negative bacteria have identical properties and have primarily been used in the previous studies of the MerR proteins^11^,^13^,^29^,^30^. Here we report the crystal structure of the C-terminally truncated *Tn501* MerR in complex with mercury(II). The crystal structure offers insights into the coordination chemistry of MerR regulators with mercury(II) and contributes to elucidating the allosteric network across the protein dimer interface for mercury(II)-binding signal transmission in Gram-negative bacteria.

## Results and Discussion

### Overall structure of mercury(II)-bound *Tn501* MerR

**Figure 1:**
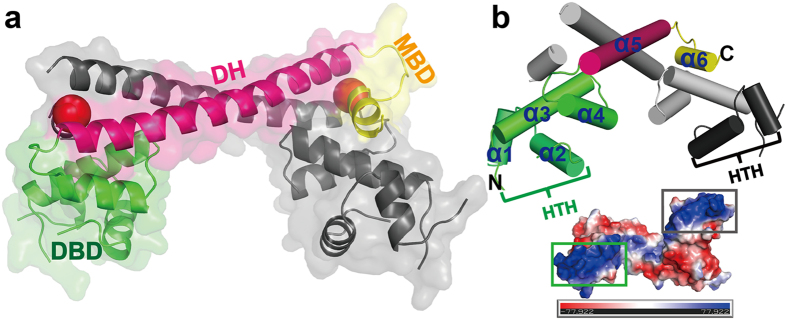
Overall structure of the mercury(II)-bound MerR dimer. (**a**) Stereoview of the overall structure of mercury(II)-bound *Tn501* MerR (PDB code 5CRL). The backbone structure of *Tn501* MerR is shown in a cartoon representation, in which one monomer is indicated in gray, and the other monomer is indicated with different colors for the three functional domains. (**b**) The topological arrangement of *Tn501* MerR and its electrostatic surface. The positively charged helix-turn-helix (HTH) motifs on the electrostatic surface are colored in blue.

The mercury(II)-bound *Tn501* MerR was crystallized as a functional dimer in an asymmetrical unit of the P3_1_21 space group. Clear electron density could be traced for the residues 6–34, 44–120, and 126–133 of monomer A and for residues 7–34 and 44–134 of monomer B. The structure was refined to 2.8 Å resolution with crystallographic *R*-factors of *R*_*work*_ = 0.230 and *R*_*free*_ = 0.266 (Table 1). The overall folding of *Tn501* MerR is similar to that of other members of the MerR-family group, such as CueR, ZntR, SoxR, BmrR, MtaN, and the *Bacillus* MerR from Gram-positive bacteria^28^,^31^,^32^,^33^,^34^,^35^,^36^,^37^,^38^,^39^. Each monomer contains three distinct functional domains comprising an N-terminal DNA-binding domain (DBD, residues 1–81), a C-terminal metal-binding domain (CBD, residues 119–134), and an amphipathic dimerization helix (DH, residues 82–118) (Fig. 1a). The functional domains are mainly constituted by α-helices, and all of them participate in the dimerization. The accessible surface area for the functional dimer is ~4000 Å^2^. The long dimerization helix plays a critical role in stabilizing the dimer by the formation of an antiparallel coiled-coil dimer interface. The circular dichroism (CD) spectra have shown that the shortened protein (residues 80–128) still retains the α-helical structure and forms a stable dimer^27^. In the DNA-binding domain, the N-terminal helix-turn-helix (HTH) motif was found to be positively charged in the electrostatic analysis, which indicates that it is suitable for interaction with the promoter DNA (Fig. 1b).

### Mercury(II)-binding domain of *Tn501* MerR

**Figure 2:**
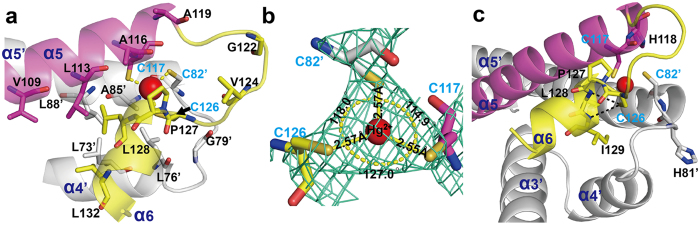
The surrounding environment of metal-binding domain in *Tn501* MerR. (**a**) Stereodrawing of the metal-binding region in *Tn501* MerR. The hydrophobic residues are shown in stick representation and are labeled in black. The coordinated cysteine residues are labeled in cyan, and the interactions with mercury(II) are indicated by a yellow dotted line. (**b**) The electron density maps of the mercury(II)-binding site in *T*n*501* MerR. The 2F_o_-F_c_ omit map contoured at the 2σ level is shown by green meshes. The Hg-S bonds are shown as yellow lines with bond lengths. The bond angles between the Hg-S bonds are shown in yellow. (**c**) The pivotal residues (His81, His118, Pro127, L128, and I129) required to maintain a suitable electrical charge for mercury(II) binding. The N-H…S hydrogen-bonding interactions are shown as black dotted lines.

In the functional dimer of *Tn501* MerR, there are two mercury ions bound at the equivalent site (Fig. 1a). The metal-binding domain comprises a flexible metal binding loop (residues 119–127) and a short two-turn helix (α6, residues 128–134), which is packed against the N-terminus of the dimerization helix and the α4′-helix of the other monomer (Fig. 2a). However, only one metal-binding loop in the MerR dimer is fully ordered (Fig. 1a). Residues 121–125 in one monomer’s metal-binding loop are disordered, possibly because of crystal packing. A similar phenomenon was found in the crystal structure of Cu(I)-CueR^31^. The flexible loop and the hydrophobic dimerization helix core constitute a closed and rigid moiety that contributes to the stabilization of mercury(II) binding (Fig. 2a).

In *Tn501* MerR, the bounded mercury(II) shows planar trigonal coordination geometry with the S-atoms from three conserved cysteine residues (Cys82, Cys117, and Cys126). This finding is consistent with the conclusions identified by ^199^Hg-NMR and extended X-ray absorption fine structure (EXAFS) studies^24^,^26^. The Hg–S bond length in the mercury(II)-cysteine complex is consistent at ~2.56 Å, and the largest S-Hg-S angle is at 127° (Fig. 2b). The angular deviation from the perfect 120° may be caused by a steric effect derived from the pyrrolidine of Pro127, which is packed above the Hg-S_Cys117_ and Hg-S_Cys126_ bonds (Fig. 2c). Cys126 defines the ending of the metal-binding loop, and its S atom is constrained by Pro127 over the center of the polar two-turn helix. This provides the S atom of Cys126 a proper position to interact with residues L128 and I129 on the polar two-turn helix^31^ (Fig. 2c). The resulting N-H…S hydrogen-bonding interactions are helpful in neutralizing the additional charge derived from the thiolate anion of residue Cys126. If the Pro127 residue is mutated (P127L), the MerR protein is impaired in mercury(II) induced transcriptional activation^13^. Of note, the sequence CPLI is conserved among the MerR proteins from Gram-negative bacteria (Supplementary Fig. S1). According to the study of CueR protein^31^, the other redundant negative charge could be neutralized by the distant charge-charge interaction between histidine residues (His81’ and His118) and coordinated ligands (Cys82′ and Cys117). The mutation of His118 to nonpolar Ala abolishes the mercury(II)-dependent transcriptional activation^40^.

### Structural differences between MerR proteins from different sources

**Figure 3:**
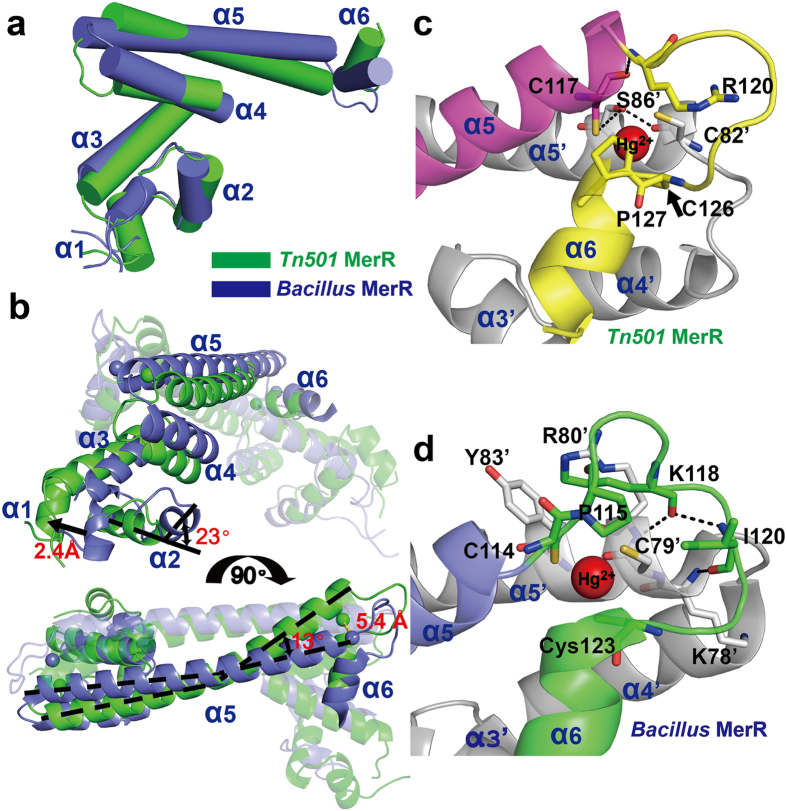
The structural alignments between mercury(II)-bound *Tn501* MerR and *Bacillus* MerR. (**a**) Tertiary structural superimpositions and (**b**) Quaternary structural superimpositions of the Hg^2+^-MerR complex between *Tn501* MerR (PDB code 5CRL) and *Bacillus* MerR (PDB code 4UA1). The structures of MerR proteins are shown as cartoon representations, with *Tn501* MerR in green and *Bacillus* MerR in slate. (**c**) A stereodrawing of the metal-binding region in *Tn501* MerR. (**d**) A stereodrawing of the metal-binding region in *Bacillus* MerR. In (**c**,**d**), the residues in the metal-binding region are shown as stick representations. The interactions within this region are shown as black dotted lines.

Despite their low sequence homology, *Tn501* MerR and *Bacillus* MerR displayed similar secondary structural arrangements. Nevertheless, the length of each secondary structural elements in the two MerR proteins was different (Supplementary Fig. S1). More importantly, in the mercury(II)-bound state, both the tertiary and quaternary structural arrangements between the two MerR proteins showed considerable differences (Fig. 3a,b). In the tertiary structural comparison (Fig. 3a), the extension directions of every structural element were different, which consequently caused greater differences in the quaternary structural comparison (Fig. 3b). A root-mean-square deviation (r.m.s.d.) of 5.34 Å for 214 structurally aligned Cα positions was produced after the quaternary structural superimposition between *Tn501* MerR and *Bacillus* MerR. Compared with *Bacillus* MerR, the quaternary structure of *Tn501* MerR showed a ~2.4 Å translation of the α1-helix and an outward rotation of approximately 23° for the α2-helix (Fig. 3b). With a small translation of the α3-helix, the α4- and α5-helices of *Tn501* MerR had a lower position than those of *Bacillus* MerR (Fig. 3b). In addition, the α5-helices of the two MerR proteins in the quaternary structural comparison had an included angle of 13°, and the mercury(II) ions in the corresponding binding site of the two MerR proteins were 5.4 Å apart (Fig. 3b).

It has been unclear why MerR proteins from different sources have differences in their tertiary and quaternary structural arrangements in the mercury(II)-bound form, although they execute the same function as the mercury(II)-responsive transcription activator. We speculate that the different spacer between the −35 and −10 elements of the promoter DNA may explain the differences. The spacer is 20 bp in Gram-positive bacteria, which is longer than that of the 19 bp spacer in Gram-negative bacteria^32^,^41^. The previous genetic analysis of the *mer* promoter in Gram-negative bacteria has shown that the insertion of 1 or 2 bp in the spacer will abolish the promoter DNA activity^42^. This indicates that the structures of MerR proteins from two different sources are tailored to their respective promoter DNA. In the studies of other MerR-family proteins, the α2-helix was defined as the DNA-recognition helix that inserts into the major groove of promoter DNA in both the repressor and activator complexes^32^,^35^,^38^. In the mercury(II)-bound *Tn501* MerR and *Bacillus* MerR, the distances between the two DNA-recognition helices are 33.4 Å and 28.6 Å, respectively (Supplement Fig. S2). This distance will not lead to obvious changes in the DNA-bond form of the activator MerR, as in the structure study of homologous CueR protein, where the structural deviations between the Ag(I)-CueR and Ag(I)-CueR-DNA were found to be relatively small^35^. Thus, the MerR proteins from Gram-positive and Gram-negative bacteria will adopt different tertiary and quaternary arrangements, particularly in the DNA-binding domain, to interact with their respective promoter DNA for transcriptional initiation.

The differences in the structural arrangement are supported by the distinct interactions within the residues. For example, in the mercury(II)-binding domain, the three conserved cysteine residues and other corresponding residues in the two MerR proteins have different interactions with the surrounding environment (Fig. 3c,d). In *Tn501* MerR, the polar residue Ser86′ forms hydrogen bonds with the O atom of Cys82′ and the S atom of Cys117 via hydroxyl oxygen. These interactions are absent in the corresponding site in *Bacillus* MerR, owing to the steric effect of residue Tyr83′. A hydrogen-bonding interaction was observed between residues Cys117 and Arg120 on the metal-binding loop in *Tn501* MerR. However, this interaction is disrupted by the rigid residue Pro115 following residue Cys114 in *Bacillus* MerR. In addition, in a comparison of their quaternary structures of the metal-binding domains, the interactions between residues Lys78 and Ile120, along with residues Arg80 and Lys118, can be considered to cause the metal-binding loop to be packed more closely to the N-terminus of the α5 helix in *Bacillus* MerR compared with *Tn501* MerR (Supplementary Fig. S3a). Despite the distinct steric effects and polar interactions contributed by the surrounding residues, the mercury(II) coordination configuration presented in *Bacillus* MerR is identical to that of *Tn501* MerR (Supplementary Fig. S3b). There are small differences in the Hg-S bond length and angle, and both of the trigonal planar Hg-thiolate configurations in the two MerR proteins are reasonable, with similar single-point energies (Supplementary Table S1), thus indicating that the trigonal planar configuration of MerR in complex with mercury(II) is common in Gram-positive and Gram-negative bacteria.

### Ultrasensitivity and heavy-metal selectivity of MerR for mercury(II)

In *Tn501* MerR, the coordinated residues Cys117 and Cys126 are separately located on the surface of the dimerization helix and the flexible metal-binding loop in positions corresponding to residues Cys112 and Cys120 in CueR (Supplementary Fig. S4)^31^. The additional coordinated ligand Cys82′ at the dimerization helix of the other monomer not only strengthens the stability of the dimer in mercury(II)-free form but also provides a higher coordination number that is optimal for mercury(II) binding. The mutant C82Y was demonstrated to be seriously affected in terms of its dimerization and mercury(II) binding affinity^22^. Although the linear dithiolate geometry is predominant in the mercury(II) complex^43^, the unusual planar trigonal coordination geometry confers the MerR protein with a higher affinity for mercury(II). In addition to Cys82, mutations of the other two coordinated cysteine residues also dramatically decreased the affinity of MerR for mercury(II)^13^,^22^,^23^. We initially attempted to determine the MerR-Hg^2+^ binding constant by UV-titration in competition experiments using small molecular mercury(II) ligands with known association constants for mercury(II). None of the competitors, including EDTA, DTPA (Diethylene Triamine Pentacetic Acid), TTHA (Triethylene Tetramine Hexaacetic Acid), or even CN^−^ at 100-fold amounts, was able to compete the mercury(II) away from MerR (Supplement Fig. S5). On the basis of the apparent equilibrium constant determined by the O’Halloran group in the competition of L-cysteine^29^,^43^, the association constant of *Tn501* MerR protein for mercury(II) was estimated to be on the order of 10^47^.

In addition, the unusual planar trigonal geometry also makes MerR distinguish Hg(II) from other metal ions. The previous studies have revealed that more than two- to three-orders of magnitude of Cd(II), Zn(II), Ag(I), Au(I) and Au(III) than Hg(II) are needed to partially stimulate transcription *in vitro*^15^. The preferred coordination geometry of different metal ions may contribute a lot to the high degree of selectivity^40^. In *de novo* designed three-helix bundle peptides, Hg(II) coordinates with tri-cysteine in a planar trigonal complex^44^,^45^. In contrast, Cd(II) and Zn(II) adopt a pseudotetrahedral geometry with three cysteine residues and an exogenous water molecule as the fourth ligand^44^,^45^. A systematic statistical analysis of the crystal structures deposited in the Protein Data Bank has revealed that Cd(II)/Zn(II)-containing proteins often adopt tetrahedral coordinated configurations^46^,^47^,^48^. When Zn(II) and Cd(II) are substituted for Hg(II) in MerR, this protein may adopt a pseudotetrahedral geometry at lower level binding constants, as in the designed peptides. For Ag(I) and Au(I), they prefer to adopt a liner coordination geometry with cysteine residues as in the Ag(I)-CueR and Au(I)-GloB complexes^31^,^49^. Other metal ions, such as Mn(II), Co(II), Ni(II), Pt(II), Cu(I), Tl(I) and Pb(II), cannot form proper or stable complexes with the purified MerR, making them unable to activate the transcription *in vitro* even at high concentrations^15^. In cells, the concentrations of metal ions and MerR protein are not as high as those used in the *in vitro* experiments. Thus, the wild-MerR responds silently *in vivo* to Ag(I), Au(I), or Zn(II) and only weakly to Cd(II)^40^. In addition, although extensive single or multiple mutations can improve the sensitivity to Cd(II), none of these variations change the preference of MerR for Hg(II)^40^.

### Allosteric signaling network in *Tn501* MerR

**Figure 4:**
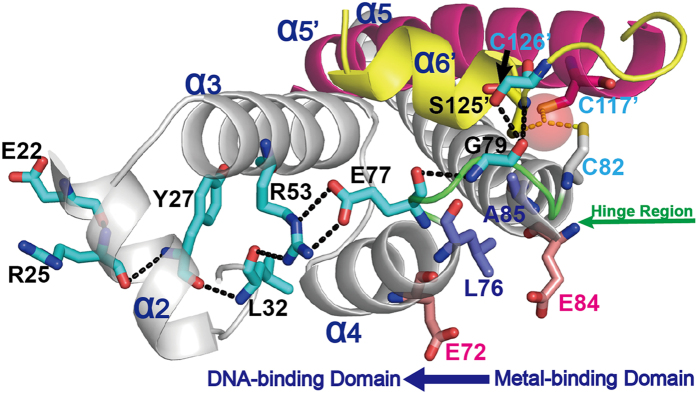
Allosteric signaling network in *Tn501* MerR. The residues in the allosteric pathway are shown in stick representation. The interactions between these residues are depicted as black dotted lines.

Upon mercury(II) binding, a series of conformational changes occur in *Bacillus* MerR, including the relocation of the metal-binding motif and a shift in the DNA-binding domain^28^. Because the same regulatory mechanism is shared by many MerR-family proteins, similar conformational changes induced by mercury(II) are likely to occur in MerR proteins from Gram-negative bacteria. A transition from a flattened compact conformation to an extended conformation was demonstrated using small-angle X-Ray scattering (SAXS) with *Tn21* MerR titrated by mercury(II)^20^. There are allosteric pathways across the dimer interface linking the metal-binding domain and the DNA-binding domain. In *E. coli* CueR, the key residue (Arg75) in the hinge region acts as an allosteric bridge^35^. Owing to the high sequence homology among MerR-family proteins, particularly in the DNA-binding domain^11^, a similar network is observed in *Tn501* MerR (Fig. 4). The Gly79 residue in the corresponding hinge region participates in hydrogen-bonding interactions not only with residues Ser125′and Cys126′ of the metal-binding domain from the other monomer, but also with residue Glu77 adjacent to the C-terminus of the α4-helix. The Oγ1 and Oγ2 atoms of residue Glu77 form a salt bridge and hydrogen-bond with the NH1 group and the Nε1 atom of residue Arg53, respectively. Residue Arg53 on the α3-helix further propagates the allosteric signal through hydrogen-bond interactions between residues Leu32 and Tyr27 on the DNA-recognition α2-helix. Upon mercury(II) binding, a large chemical shift at Tyr27 occurs, as has been observed by ^19^F-NMR^50^.

In addition to the three coordinated cysteine residues, the results of previous mutagenesis analyses have indicated that mutations near or at the aforementioned amino acids also significantly influence the repressive or responsive functions of *Tn501* and *Tn21* MerR. The repressive function is influenced by the mutation of residues Ser125 (S125P), Gly79 (G79S), and Arg53 (R53W and R53Q)^40^. Other mutants (E72K, L76F, E84K, and A85V) around residue Gly79 on the hinge region (Fig. 4) are also repression-deficient^30^,^51^,^52^. The mutation of residues Glu72 and Glu84 to the Lys (E84K or E72K) induces electrovalent-bond and hydrogen-bond interactions, and these newly formed interactions favor the activated conformation in the absence of mercury(II)^30^,^51^,^52^. Similar results have been reported for residues Ala85 and Leu76. The mutations A85V and L76F also promote the formation of hydrophobic interactions that activate transcription in the absence of mercury(II)^30^,^51^,^52^. The single mutant E77K shows strong mercury(II)-independent activation, and this activity is enhanced in the double mutant A89V-E77K as well as the triple mutant A89V-D78N-E77K^40^. In the DNA-binding domain, mutations in residue Tyr27, as well as the nearby residues Glu22 and Arg25, yield proteins deficient in promoter DNA binding^22^,^50^,^51^. The activation and repression activities conferred by the E22K and R25H mutants are much lower than observed for the wide-type protein^22^,^51^. Based on the structural information obtained from the characterized protein-DNA complex and the sequential alignments in the MerR-family proteins^32^,^35^,^38^, Glu22 is thought to be the residue involved in base-direct contacts that confers the DNA-recognition specificity, and Arg25 interacts with the DNA back-bone. These experiments further indicate that the hypersensitivity of MerR for mercury(II) relies not only on the coordinated residues, but also on the sophisticated cooperation of residues from all functional domains.

Although the aforementioned residues in *Tn501* MerR are identical or conserved substitutions among the MerR from Gram-negative bacteria, most of these amino acids exhibit substantial differences compared to MerR from Gram-positive bacteria^11^ (Supplementary Fig. S1). Interestingly, when some residues in Gram-negative bacteria are replaced with the corresponding residues in Gram-positive bacteria, such as the E72K and E77K mutants, a functional deficiency in transcription occurs^30^,^40^. All of these findings indicate that, in the MerR proteins from Gram-negative and Gram-positive bacteria, different allosteric networks with distinct tertiary and quaternary structural arrangements are adopted to propagate the mercury(II)-binding signal.

## Conclusions

In this work, the crystal structure of *Tn501* MerR from Gram-negative bacteria is presented in the mercury(II)-bound form. It displays an appropriate structural arrangement for divalent mercury binding in a planar trigonal configuration. The planar trigonal coordinated geometry not only provides MerR with an extremely high affinity for mercury(II) but it also allows MerR to distinguish mercury(II) from other metal ions. The hypersensitivity of MerR for mercury(II) also depends on the allosteric signaling network resulting from the cooperation of all of the functional domains. The destruction of the allosteric pathway owing to a mutation of any participant residue will affect the transcriptional repression, the transcriptional activation, or both, of MerR. With the high sequence homology, the crystal structure of *Tn501* MerR provides insights into the functions of MerR proteins from Gram-negative bacteria. More interestingly, it shows significant differences with regard to the tertiary and quaternary structural arrangements of the *Bacillus* MerR from Gram-positive bacteria, at least in the mercury(II)-bound form. The MerR proteins from two different sources arrange their structures differently, probably to allow them to function with promoter DNA with different spacers between −35 and −10 elements. The crystal structure of DNA-bound repressor and activator are need to be determined and compared to provide further details regarding how the MerR proteins from different sources twist the promoter DNA and initiate transcription.

## Materials and Methods

### Construction, expression, and purification of *Tn501* MerR

The synthesized and optimized *merR* gene was PCR amplified and cloned into the pET-30a vector between the NdeI and XhoI sites. *E. coli* BL21 (*DE3*) cells containing the constructs were grown in LB medium supplemented with 30 μg/mL of kanamycin. The cells were cultivated at 37 °C with constant shaking at 250 rpm. Expression was induced by adding 0.5 mM IPTG once an optical density of OD 600 ≈ 0.6 was reached, and growth continued for an additional 4 h at 30 °C.

The cells were harvested by centrifugation and were resuspended in 15 ml of lysis buffer [20 mM sodium citrate, pH 6.0, 20 mM ammonium tartrate, 20 mM NaCl, 10 mM β-ME, 0.1 mM PMSF, 5% glycerol, and 1 μL of DNaseI]. The suspension was disintegrated by sonication on ice, and the supernatant was removed by centrifugation at 12000 rpm for 15 min. The pellet was washed twice with 20 ml of buffer A [20 mM sodium citrate, pH 6.0, 20 mM ammonium tartrate, and 10 mM β-ME] and resuspended in 20 ml of buffer B [0.7 M (NH_4_)_2_SO_4_ in buffer A]. Subsequently, the pellet was incubated with buffer B at 4 °C for 30 min. The resuspended MerR protein was obtained by centrifugation at 12000 rpm for 15 min and was loaded onto a 5-ml HiTrap Phenyl FF column pre-equilibrated with buffer B. After being washed with 5–6 column volumes of buffer B, the protein was eluted with buffer A containing 0.4 M (NH_4_)_2_SO_4_ and was subsequently applied to a 5-ml HisTrap HF. The MerR protein without a His-Tag showed weak affinity for the Ni-NTA column and was eluted with 25 mM imidazole and 500 mM NaCl in buffer A. The fractions containing MerR were concentrated and injected onto a gel filtration column (HiLoad 26/60 Superdex200, GE Healthcare) equilibrated with buffer C [20 mM sodium citrate, pH 6.0, 20 mM ammonium tartrate, 250 mM NaCl, and 0.5 mM TCEP]. Peak fractions containing the MerR dimer were collected and concentrated for further experiments.

The expression and purification of C-terminal truncated *Tn501*MerR (MerRTC10, the last ten C-terminal residues of *Tn501*MerR were truncated) was performed in the same manner.

### Crystallization

To crystallize Hg^2+^-bound *Tn501* MerR, purified MerR protein (4–5 mg/ml) was mixed with an equimolar amount of HgCl_2_. Crystals of the full-length Hg^2+^-MerR complex were grown at 16 °C by the sitting-drop vapor-diffusion method against reservoirs containing 1.5 M Li_2_SO_4_ and 0.1 M Tris pH 8.5. The C-terminal truncated MerR in complex with mercury(II) was grown in the same manner, except that the reservoir solution was 0.1 M KCl, 0.01 M MgCl_2_, 0.1 M Tris pH 8.5 and 30% PEG200. Rod-like crystals appeared 15 days later and continued to grow until reaching a suitable size for X-ray diffraction studies. The crystals were briefly soaked in a cryoprotectant containing 0.1 M KCl, 0.01 M MgCl_2_, 0.1 M Tris pH 8.5 and 38% PEG200 prior to flash-freezing in liquid nitrogen.

### Structure determination and refinement

The diffraction datasets at the mercury L3-edge were collected from single crystals at beamline BL17U of the Shanghai Synchrotron Radiation Facility and were processed using the HKL2000 software package^53^. The full-length Hg^2+^-MerR structure was diffracted to 3.7 Å but didn’t meet the standard for analysis. A trial of ten residues truncated at the C-terminal greatly improved the resolution of the crystal. The initial phase for automated model building of the truncated Hg^2+^-MerR crystal structure was solved by mercury single-wavelength anomalous dispersion (SAD) at 2.8 Å using Phenix software^54^. Iterative rounds of refinement were performed by using the Refmac software^55^, followed by manual alterations using the WinCoot software^56^. Refinement was conducted until no significant improvement was achieved. All structural models in the present study were generated with the PyMOL software^57^. The data collection and refinement statistics are listed in Table 1.

### Density functional theory calculations

The density functional theory (DFT) calculations were implemented in the Gaussian 09 package^58^. Single point energies were calculated for the crystal structures using M06-2X functional. Two types of basis sets were adopted: the standard 6–31G (d, p) basis set for nonmetal atoms and the Stuttgart-Dresden-Bonn quasirelativistic pseudopotentials (SDD) for the mercury atom. The solvent effect was considered through DFT calculations based on the implicit polarizable continuum model (PCM).

## Additional Information

**Accession codes**: The atomic coordinates and structure factors for Hg^2+^-MerR have been deposited in the Protein Data Bank (accession code 5CRL).

**How to cite this article**: Wang, D. *et al*. Structural Analysis of the Hg(II)-Regulatory Protein *Tn501* MerR from *Pseudomonas aeruginosa. Sci. Rep.* **6**, 33391; doi: 10.1038/srep33391 (2016).

## Supplementary Material

Supplementary Information

## Figures and Tables

**Table 1:** Data collection and refinement statistics for Hg^2+^-MerR.

Diffraction data	
**X-Ray source**	Synchrotron
**Wavelength/Å**	1.0069
**Detector**	ADSC QUANTUM 315r CCD
**Diffraction conditions**	100 K under N_2_ cryostream
Space group	*P* 3_1_ 2 1
Cell constants	75.01 Å 75.01 Å 97.76 Å
a, b, c, α, β, γ	90.00° 90.00° 120.00°
Resolution (Å)	30−2.7 (2.8−2.7)*
R_sym_ or R_emerge_	0.077 (0.954)
% Data completeness (in resolution range)	98.4 (98.9)
<I/σ(I)>	31.05 (1.60)
Redundancy	3.8 (3.9)
**Refinement**	
Refinement program	REFMAC 5.7.0029
Resolution (Å)	29.77–2.80
No. unique reflections	7531
R _work_/R _free_	0.230/0.266
No. atoms	
Protein	1810
Ligand	2
Water	1
B-factors	
Protein	93.570
Ligand	127.185
Water	68.010
R.m.s. deviations from ideality	
Bond lengths/Å	0.007
Bond angles/◦	1.033
Ramachandran plot**	
Favored (%)	98.2%
Allowed (%)	1.8%
Disallowed (%)	0
**Protein Data Bank code**	5CRL

^*^The highest-resolution shell is shown in parentheses.

^**^Ramachandran plot was calculated using RAMPAGE in the CCP4 suite.

## References

[b1] Tchounwou P. B., Ayensu W. K., Ninashvili N. & Sutton D. Environmental exposure to mercury and its toxicopathologic implications for public health. Environ Toxicol 18, 149–175, doi: 10.1002/tox.10116 (2003). 12740802

[b2] Silver S. & Phung le T. A bacterial view of the periodic table: genes and proteins for toxic inorganic ions. J Ind Microbiol Biotechnol 32, 587–605, doi: 10.1007/s10295-005-0019-6 (2005). 16133099

[b3] Barrineau P. . The DNA sequence of the mercury resistance operon of the IncFII plasmid NR1. J Mol Appl Genet 2, 601–619 (1983). 6530603

[b4] Stanisich V., Bennett P. & Richmond M. Characterization of a translocation unit encoding resistance to mercuric ions that occurs on a nonconjugative plasmid in Pseudomonas aeruginosa. J Bacteriol 129, 1227–1233 (1977). 403173 10.1128/jb.129.3.1227-1233.1977 PMC235085

[b5] Condee C. W. & Summers A. O. A mer-lux transcriptional fusion for real-time examination of *in vivo* gene expression kinetics and promoter response to altered superhelicity. J Bacteriol 174, 8094–8101 (1992). 1334070 10.1128/jb.174.24.8094-8101.1992 PMC207548

[b6] Misra T. K. Bacterial resistances to inorganic mercury salts and organomercurials. Plasmid 27, 4–16 (1992). 1311113 10.1016/0147-619x(92)90002-r

[b7] Barkay T., Miller S. M. & Summers A. O. Bacterial mercury resistance from atoms to ecosystems. FEMS Microbiol Rev 27, 355–384 (2003). 12829275 10.1016/S0168-6445(03)00046-9

[b8] Summers A. O. Organization, expression, and evolution of genes for mercury resistance. Annual Reviews in Microbiology 40, 607–634 (1986). 10.1146/annurev.mi.40.100186.003135 3535655

[b9] Lund P. A. & Brown N. L. Regulation of transcription in Escherichia coli from the mer and merR promoters in the transposon Tn501. J Mol Biol 205, 343–353 (1989). 2538625 10.1016/0022-2836(89)90345-8

[b10] O’Halloran T. V., Frantz B., Shin M. K., Ralston D. M. & Wright J. G. The MerR heavy metal receptor mediates positive activation in a topologically novel transcription complex. Cell 56, 119–129 (1989). 2910495 10.1016/0092-8674(89)90990-2

[b11] Brown N. L., Stoyanov J. V., Kidd S. P. & Hobman J. L. The MerR family of transcriptional regulators. FEMS Microbiol Rev 27, 145–163, doi: 10.1016/s0168-6445(03)00051-2 (2003). 12829265

[b12] Heltzel A., Lee I. W., Totis P. A. & Summers A. O. Activator-dependent preinduction binding of. sigma.-70 RNA polymerase at the metal-regulated mer promoter. Biochemistry 29, 9572–9584 (1990). 2176850 10.1021/bi00493a011

[b13] Livrelli V., Lee I. & Summers A. *In vivo* DNA-protein interactions at the divergent mercury resistance (mer) promoters. I. Metalloregulatory protein MerR mutants. J Biol Chem 268, 2623–2631 (1993). 8428939

[b14] Lund P. A., Ford S. J. & Brown N. L. Transcriptional regulation of the mercury-resistance genes of transposon Tn501. J Gen Microbiol 132, 465–480, doi: 10.1099/00221287-132-2-465 (1986). 3011964

[b15] Ralston D. M. & O’Halloran T. V. Ultrasensitivity and heavy-metal selectivity of the allosterically modulated MerR transcription complex. Proc Natl Acad Sci USA 87, 3846–3850 (1990). 2187194 10.1073/pnas.87.10.3846 PMC54000

[b16] Ansari A. Z., Bradner J. E. & O’Halloran T. V. DNA-bend modulation in a repressor-to-activator switching mechanism. Nature 374, 371–375, doi: 10.1038/374370a0 (1995). 7885478

[b17] Ansari A. Z., Chael M. L. & O’Halloran T. V. Allosteric underwinding of DNA is a critical step in positive control of transcription by Hg-MerR. Nature 355, 87–89 (1992). 1731201 10.1038/355087a0

[b18] Fleissner G., Reigle M. D., O’Halloran T. V. & Spiro T. G. UVRR Spectroscopy of the Metal Receptor Site in MerR. J Am Chem Soc 120, 12690–12691 (1998).

[b19] Frantz B. & O’Halloran T. V. DNA distortion accompanies transcriptional activation by the metal-responsive gene-regulatory protein MerR. Biochemistry 29, 4747–4751 (1990). 2364056 10.1021/bi00472a001

[b20] Guo H. B. . Structure and conformational dynamics of the metalloregulator MerR upon binding of Hg(II). J Mol Biol 398, 555–568, doi: 10.1016/j.jmb.2010.03.020 (2010). 20303978

[b21] Helmann J. D., Ballard B. T. & Walsh C. T. The MerR metalloregulatory protein binds mercuric ion as a tricoordinate, metal-bridged dimer. Science 247, 946–948 (1990). 2305262 10.1126/science.2305262

[b22] Shewchuk L. M. . Transcriptional switching by the MerR protein: activation and repression mutants implicate distinct DNA and mercury (II) binding domains. Biochemistry 28, 2340–2344 (1989). 2497778 10.1021/bi00431a053

[b23] Shewchuk L. M., Verdine G. L., Nash H. & Walsh C. T. Mutagenesis of the cysteines in the metalloregulatory protein MerR indicates that a metal-bridged dimer activates transcription. Biochemistry 28, 6140–6145 (1989). 2551364 10.1021/bi00441a002

[b24] Utschig L. M., Bryson J. W. & Halloran T. V. Mercury-199 NMR of the metal receptor site in MerR and its protein-DNA complex. Science 268, 380–385 (1995). 7716541 10.1126/science.7716541

[b25] Watton S. P. . Trigonal mercuric complex of an aliphatic thiolate: A spectroscopic and structural model for the receptor site in the mercury (II) biosensor MerR. J Am Chem Soc 112, 2824–2826 (1990).

[b26] Wright J. G., Tsang H. T., Penner-Hahn J. E. & O’Halloran T. V. Coordination chemistry of the Hg-MerR metalloregulatory protein: evidence for a novel tridentate mercury-cysteine receptor site. J Am Chem Soc 112, 2434–2435 (1990).

[b27] Zeng Q., Stålhandske C., Anderson M. C., Scott R. A. & Summers A. O. The core metal-recognition domain of MerR. Biochemistry 37, 15885–15895 (1998). 9843394 10.1021/bi9817562

[b28] Chang C. C., Lin L. Y., Zou X. W., Huang C. C. & Chan N. L. Structural basis of the mercury(II)-mediated conformational switching of the dual-function transcriptional regulator MerR. Nucleic Acids Res 43, 7612–7623, doi: 10.1093/nar/gkv681 (2015). 26150423 PMC4551924

[b29] Parkhill J., Ansari A. Z., Wright J. G., Brown N. L. & O’halloran T. Construction and characterization of a mercury-independent MerR activator (MerRAC): transcriptional activation in the absence of Hg (II) is accompanied by DNA distortion. EMBO J. 12, 413–421 (1993). 8440234 10.1002/j.1460-2075.1993.tb05673.x PMC413224

[b30] Comess K. M., Shewchuk L. M., Ivanetich K. & Walsh C. T. Construction of a synthetic gene for the metalloregulatory protein MerR and analysis of regionally mutated proteins for transcriptional regulation. Biochemistry 33, 4175–4186 (1994). 8155633 10.1021/bi00180a010

[b31] Changela A. . Molecular basis of metal-ion selectivity and zeptomolar sensitivity by CueR. Science 301, 1383–1387 (2003). 12958362 10.1126/science.1085950

[b32] Watanabe S., Kita A., Kobayashi K. & Miki K. Crystal structure of the [2Fe-2S] oxidative-stress sensor SoxR bound to DNA. Proc Natl Acad Sci USA 105, 4121–4126, doi: 10.1073/pnas.0709188105 (2008). 18334645 PMC2393793

[b33] Bachas S., Eginton C., Gunio D. & Wade H. Structural contributions to multidrug recognition in the multidrug resistance (MDR) gene regulator, BmrR. Proc Natl Acad Sci USA 108, 11046–11051, doi: 10.1073/pnas.1104850108 (2011). 21690368 PMC3131311

[b34] Kumaraswami M., Newberry K. J. & Brennan R. G. Conformational plasticity of the coiled-coil domain of BmrR is required for bmr operator binding: the structure of unliganded BmrR. J Mol Biol 398, 264–275 (2010). 20230832 10.1016/j.jmb.2010.03.011 PMC2856848

[b35] Philips S. J. . Allosteric transcriptional regulation via changes in the overall topology of the core promoter. Science 349, 877–881 (2015). 26293965 10.1126/science.aaa9809 PMC4617686

[b36] Newberry K. J. . Structures of BmrR-drug complexes reveal a rigid multidrug binding pocket and transcription activation through tyrosine expulsion. J Biol Chem 283, 26795–26804 (2008). 18658145 10.1074/jbc.M804191200 PMC2546531

[b37] Newberry K. J. & Brennan R. G. The structural mechanism for transcription activation by MerR family member multidrug transporter activation, N terminus. J Biol Chem 279, 20356–20362, doi: 10.1074/jbc.M400960200 (2004). 14985361

[b38] Heldwein E. E. Z. & Brennan R. G. Crystal structure of the transcription activator BmrR bound to DNA and a drug. Nature 409, 378–382 (2001). 11201751 10.1038/35053138

[b39] Godsey M. H., Baranova N. N., Neyfakh A. A. & Brennan R. G. Crystal structure of MtaN, a global multidrug transporter gene activator. J Biol Chem 276, 47178–47184, doi: 10.1074/jbc.M105819200 (2001). 11581256

[b40] Caguiat J. J., Watson A. L. & Summers A. O. Cd(II)-responsive and constitutive mutants implicate a novel domain in MerR. J Bacteriol 181, 3462–3471 (1999). 10348859 10.1128/jb.181.11.3462-3471.1999 PMC93814

[b41] Summers A. O. Damage control: regulating defenses against toxic metals and metalloids. Current opinion in microbiology 12, 138–144 (2009). 19282236 10.1016/j.mib.2009.02.003

[b42] Parkhill J. & Brown N. L. Site-specific insertion and deletion mutants in the mer promoter-operator region of Tn501; the nineteen base-pair spacer is essential for normal induction of the promoter by MerR. Nucleic Acids Res 18, 5157–5162 (1990). 2169606 10.1093/nar/18.17.5157 PMC332137

[b43] Wright J. G., Natan M. J., MacDonnel F. M., Ralston D. M. & O’Halloran T. V. Mercury (II)—Thiolate Chemistry and the Mechanism of the Heavy Metal Biosensor MerR. Prog Inorg Chem 38, 323–412 (1990).

[b44] Chakraborty S. . Design of a Three‐Helix Bundle Capable of Binding Heavy Metals in a Triscysteine Environment. Angew Chem Int Ed 50, 2049–2053 (2011). 10.1002/anie.201006413 PMC3058785 21344549

[b45] Mocny C. S. & Pecoraro V. L. De Novo Protein Design as a Methodology for Synthetic Bioinorganic Chemistry. Acc Chem Res 48, 2388–2396 (2015). 26237119 10.1021/acs.accounts.5b00175 PMC5257248

[b46] Dokmanić I., Šikić M. & Tomić S. Metals in proteins: correlation between the metal-ion type, coordination number and the amino-acid residues involved in the coordination. Acta Crystallogr D Biol Crystallogr 64, 257–263 (2008). 18323620 10.1107/S090744490706595X

[b47] Laitaoja M., Valjakka J. & Janis J. Zinc coordination spheres in protein structures. Inorg Chem 52, 10983–10991, doi: 10.1021/ic401072d (2013). 24059258

[b48] Rulíšek L.r. & Vondrášek J. Coordination geometries of selected transition metal ions (Co^2+^, Ni^2+^, Cu^2+^, Zn^2+^, Cd^2+^, and Hg^2+^) in metalloproteins. J Inorg Biochem 71, 115–127 (1998). 9833317 10.1016/s0162-0134(98)10042-9

[b49] Wei W. . Structural Insights and the Surprisingly Low Mechanical Stability of the Au–S Bond in the Gold-Specific Protein GolB. J Am Chem Soc 137, 15358–15361 (2015). 26636614 10.1021/jacs.5b09895

[b50] Song L., Teng Q., Phillips R. S., Brewer J. M. & Summers A. O. 19F-NMR reveals metal and operator-induced allostery in MerR. J Mol Biol 371, 79–92, doi: 10.1016/j.jmb.2007.04.085 (2007). 17560604

[b51] Parkhill J., Lawley B., Hobman J. L. & Brown N. L. Selection and characterization of mercury-independent activation mutants of the Tn501 transcriptional regulator, MerR. Microbiology 144, 2855–2864 (1998). 9802027 10.1099/00221287-144-10-2855

[b52] Ross W., Park S. J. & Summers A. O. Genetic analysis of transcriptional activation and repression in the Tn21 mer operon. J Bacteriol 171, 4009–4018 (1989). 2661542 10.1128/jb.171.7.4009-4018.1989 PMC210155

[b53] Otwinowski Z. & Minor W. [20] Processing of X-ray diffraction data collected in oscillation mode. Methods Enzymol 276, 307–326 (1997). 10.1016/S0076-6879(97)76066-X 27754618

[b54] Adams P. D. . PHENIX: a comprehensive Python-based system for macromolecular structure solution. Acta Crystallogr D Biol Crystallogr 66, 213–221, doi: 10.1107/S0907444909052925 (2010). 20124702 PMC2815670

[b55] Murshudov G. N., Vagin A. A. & Dodson E. J. Refinement of macromolecular structures by the maximum-likelihood method. Acta Crystallogr D Biol Crystallogr 53, 240–255, doi: 10.1107/S0907444996012255 (1997). 15299926

[b56] Emsley P., Lohkamp B., Scott W. G. & Cowtan K. Features and development of Coot. Acta Crystallogr D Biol Crystallogr 66, 486–501, doi: 10.1107/S0907444910007493 (2010). 20383002 PMC2852313

[b57] DeLano W. L. PyMOL. DeLano Scientific, San Carlos, CA 700 (2002).

[b58] Frisch M. . Gaussian 09, Revision A. 02, Gaussian. Inc., Wallingford, CT 200 (2009).

