# Supplementary material for: Structural Analysis of the Hg(II)-Regulatory Protein Tn501 MerR from Pseudomonas aeruginosa
Source: Sci Rep. 2016 Sep 19;6:33391. doi: 10.1038/srep33391 (PMC5027573; doi:10.1038/srep33391)
Supplement: Supplementary Information [file srep33391-s1.doc]

**Supporting Information for:**

Structural Analysis of the Hg (II)-Regulatory Protein

*Tn501* MerR from *Pseudomonas aeruginosa*

Dan Wang^1^, Shanqing Huang^1^, Pingying Liu^2^, Xichun Liu^1^, Yafeng He^1^, Weizhong Chen^1,3^, Qingyuan Hu^1^, Tianbiao Wei^1^, Jianhua Gan^4^, Jing Ma*^,2^, and Hao Chen*^,1^

^1^Coordination Chemistry Institute and the State Key Laboratory of Coordination Chemistry, School of Chemistry and Chemical Engineering, Collaborative Innovation Center of Chemistry for Life Sciences, Nanjing University, Nanjing 210093, P. R. China;

^2^Institute of Theoretical and Computational Chemistry, School of Chemistry and Chemical Engineering, Nanjing University, Nanjing 210093, P. R. China;

^3^Department of Chemical Physics, University of Science and Technology of China, Hefei 230026, P. R. China;

^4^School of Life Sciences, Fudan University, Shanghai 200433, P. R. China.

**Correspondence to Jing Ma and Hao Chen:** [majing@nju.edu.cn](mailto:majing@nju.edu.cn); chenhao@nju.edu.cn

**This file contains:**

**- Supplementary** Figures S1-S5

**- Supplementary** Table S1

**- Supplementary** Materials and Methods

**- Supplementary** References

**
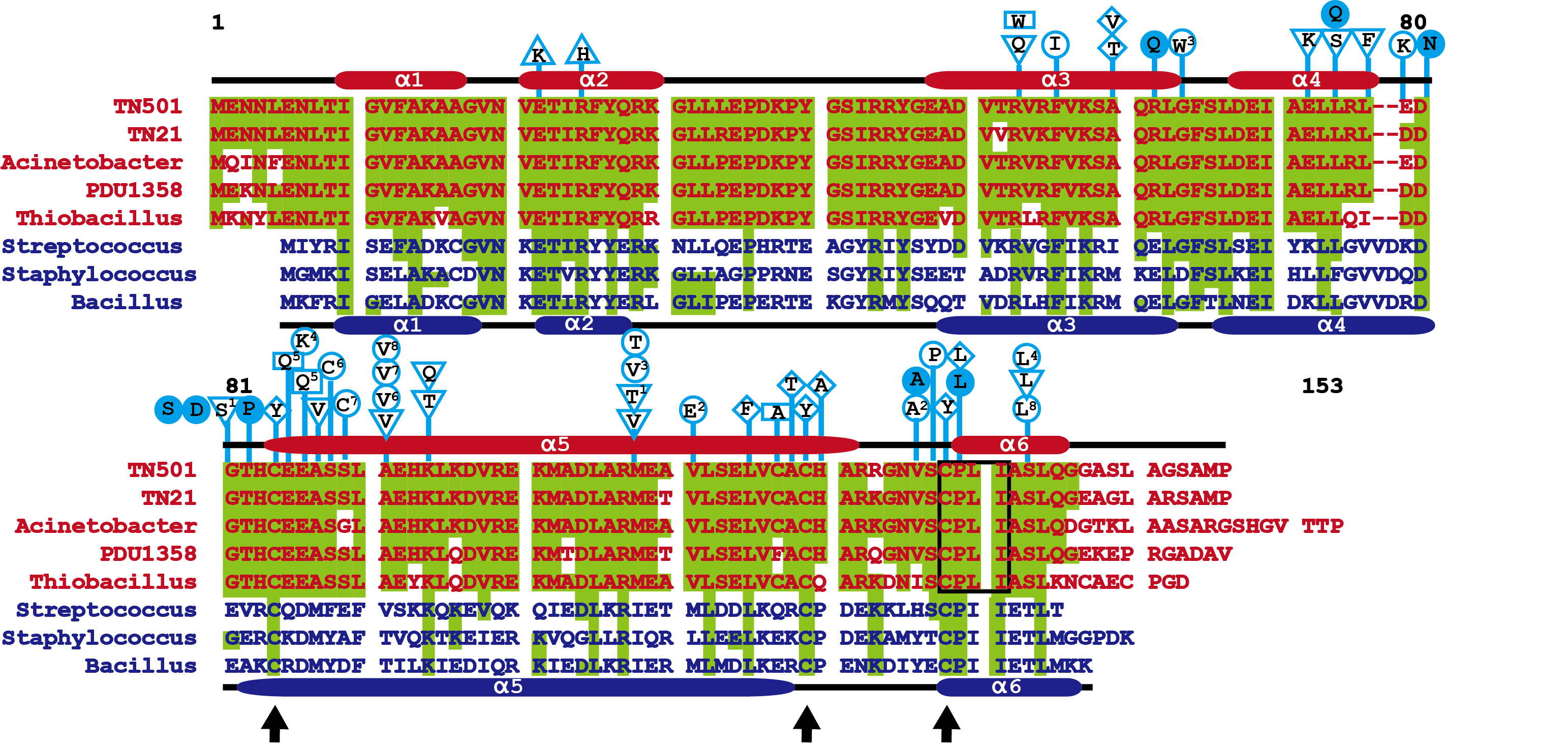
**

**Figure S1.** Sequence alignment of MerR proteins from a variety of Gram-negative and Gram-positive sources. The sequences of MerR proteins from Gram-negative bacteria are shown in red, and those from Gram-positive bacteria are shown in blue. The conserved amino acids between Gram-negative and Gram-positive bacteria (approximately 22%, 32 residues) are shaded with green shadows. The mercury(II)-binding cysteine residues are indicated with black arrows. Mutations performed in Gram-negative bacteria are shown above the amino acid sequences^1^. (Symbols: □, repression-deficient only; ▽, Cd(II) responsive; ○, fully constitutive; ◊, activation; ∆, activation- and repression-deficient; ●, constitutive mutants constructed in an A89V and S131L background). The conserved sequence CPLI on the two-turn helix in Gram-negative bacterial is indicated with a black box. Secondary structural arrangements for Gram-negative bacteria are shown above the sequences according to that of *Tn501* MerR, and secondary structural arrangements for Gram-positive bacteria are shown below the sequences according to that of *Bacillus* MerR. Sequence alignment was performed using Multiple^2^. The NCBI accession numbers are: **CAA77320** (*Tn501* MerR), **P0A2Q9** (*Tn21* MerR), **WP_005136947** (*Acinetobacter baumannii*), **AAA98221** (*PDU1358*), **CAA72395** (*Thiobacillus spp.*), **ALP88439** (*Streptococcus agalactiae*), **KFL05890** (*Staphylococcus aureus*), and **CAA71041** (*Bacillus megaterium*).


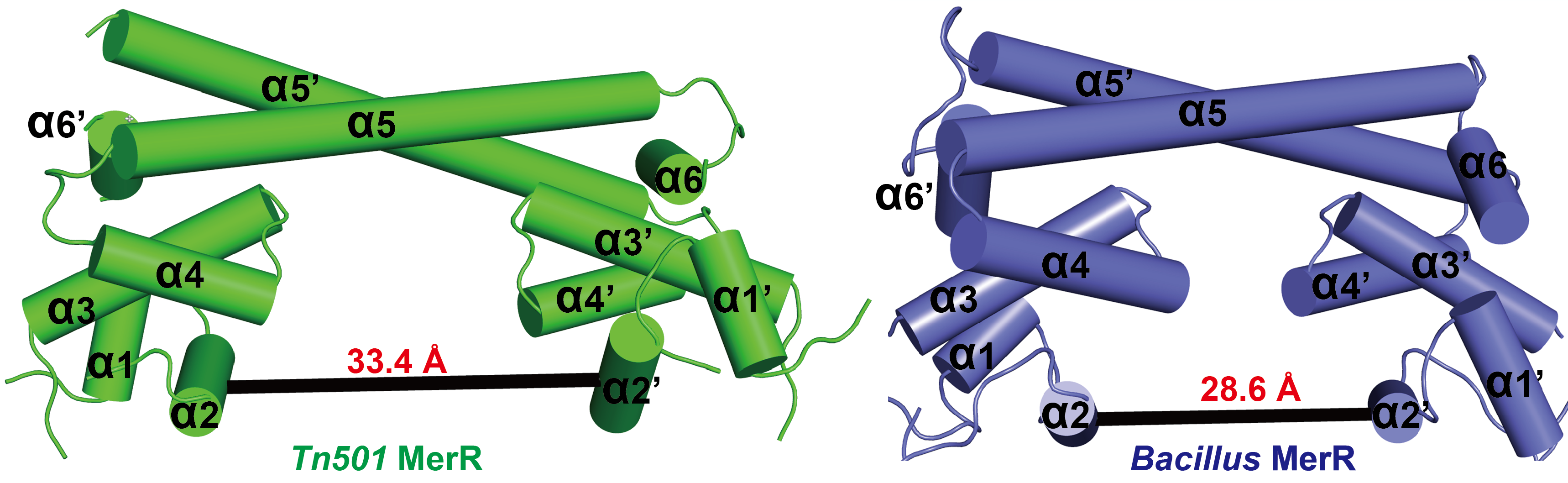


**Figure S2.** Distance between the recognition helices (α2 and α2’-helices) in the mercury(II)-bound *Tn501* MerR and *Bacillus* MerR. The structures are shown as cartoon representation with *Tn501* MerR colored in green and *Bacillus* MerR colored in slate.


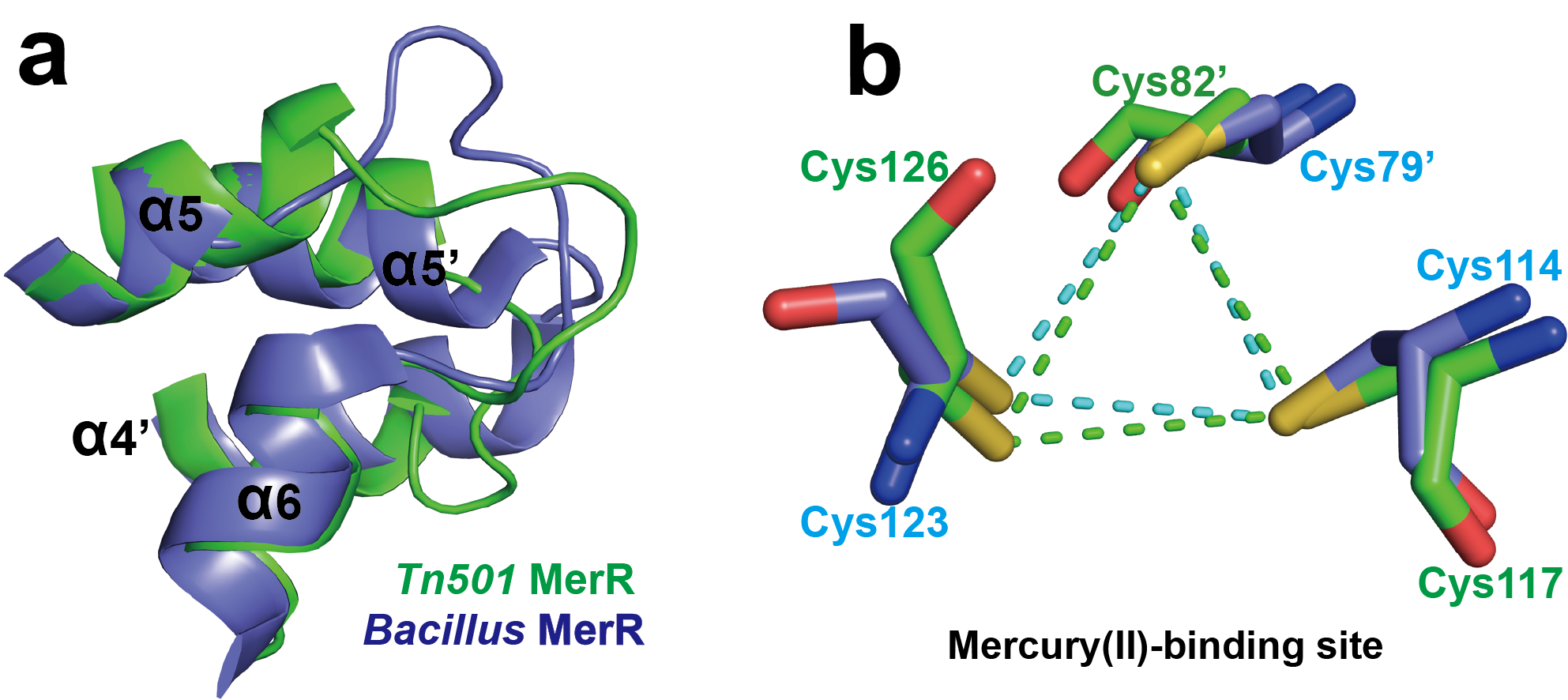


**Figure S3.** (a) Quaternary structural alignment of the mercury(II)-binding domains between *Tn501* MerR and *Bacillus* MerR (PDB code 4UA1)^3^. The structures are shown as cartoon representation with *Tn501* MerR colored in green and *Bacillus* MerR colored in slate. (b) The structural alignment of the coordinated cysteine residues between *Tn501* MerR and *Bacillus* MerR. The cysteine residues are shown as stick representations, with O atoms in red, N atoms in blue and S atoms in yellow. The backbone C atoms in *Tn501* MerR are green, and those in *Bacillus* MerR are slate.


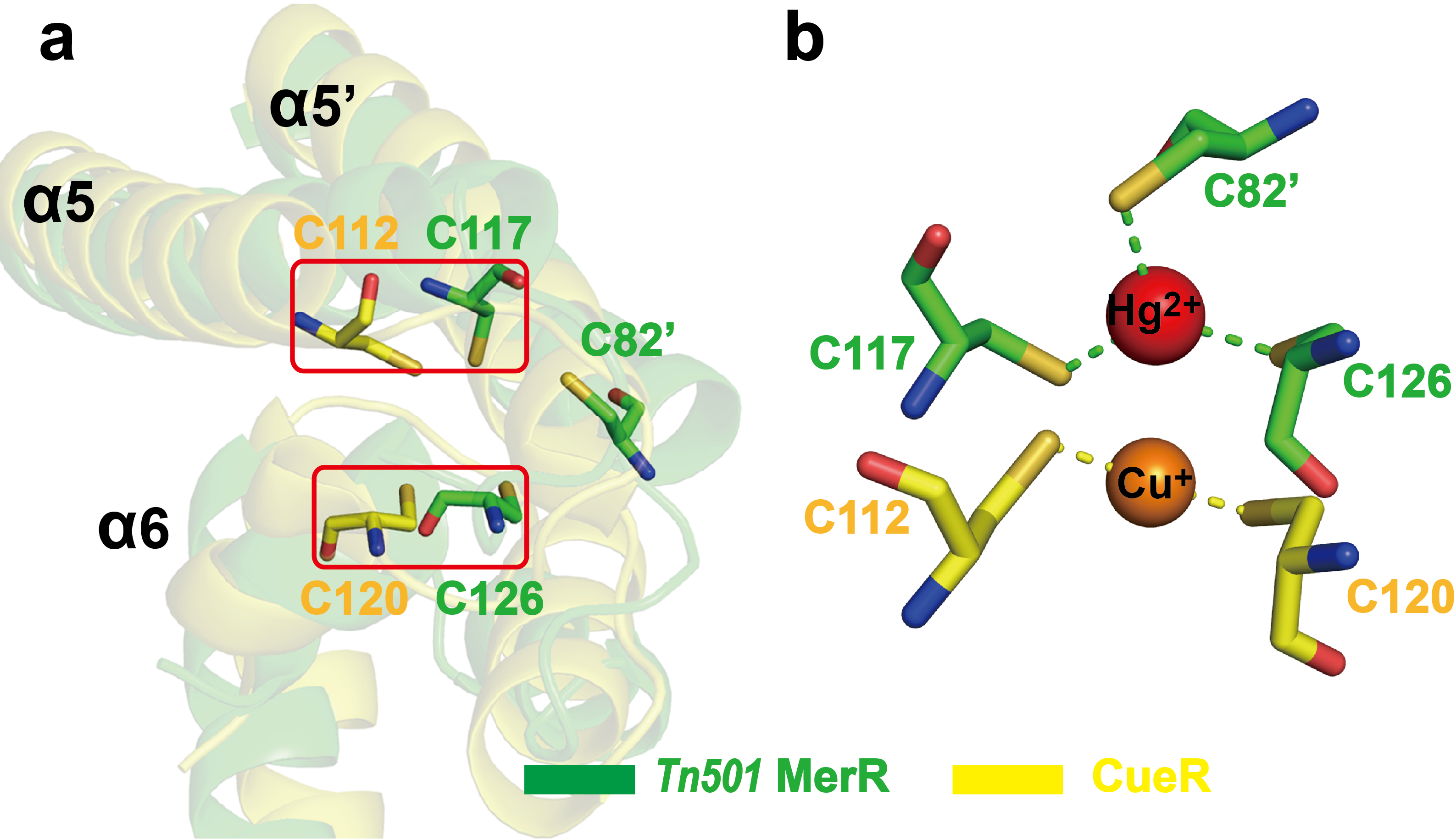


**Figure S4.** (a) Quaternary structure alignment of the metal-binding domains between *Tn501* MerR and CueR (PDB code 1Q05)^4^. The structures are shown as cartoon representations, with *Tn501* MerR in green and CueR indicated in yellow. (b) The structural alignment of coordinated cysteine residues between *Tn501* MerR and CueR. The cysteine residues are shown as stick representations, with O atoms in red, N atoms in blue and S atoms in yellow. The backbone C atoms in *Tn501* MerR are green, and those in CueR are yellow.


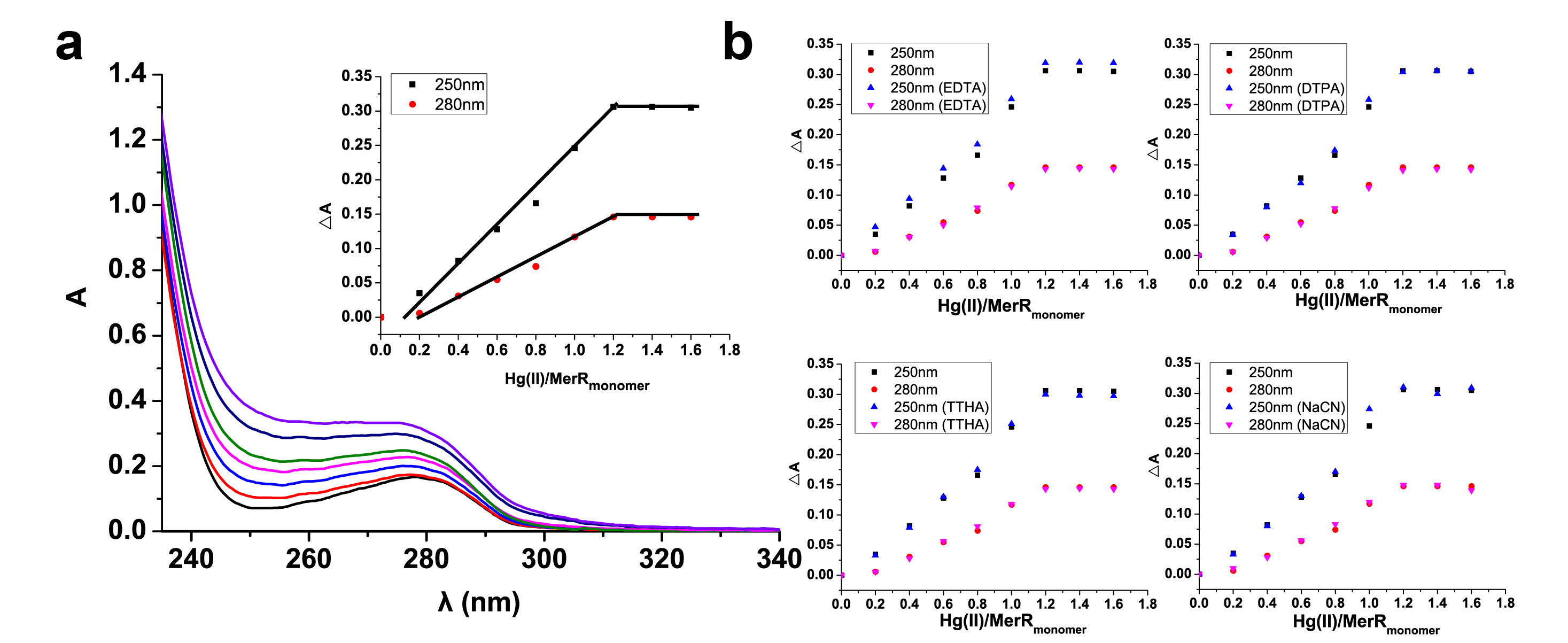


**Figure S5.** (a) UV spectra of the Hg(II)-MerR complex. Spectra were obtained by adding different concentrations of HgCl_2_ into 38×10^-6^ M protein solution in 20 mM sodium citrate, pH 6.0, 20 mM ammonium tartrate, 250 mM NaCl, and 0.5 mM TCEP. Spectra in different colors represent the addition of 0, 0.2, 0.4, 0.6, 0.8, 1.0, and 1.2 equiv of Hg(II)/MerR_monomer_. Inset: A plot of the absorbance changes at 250 nm and 280 nm versus the ratio of Hg(II) to the MerR monomer. (b) A plot of the absorbance changes at 250 nm and 280 nm versus the ratio of Hg(II) to the MerR protein incubated with and without different competitors. (38×10^-6^ M MerR protein solution, 250×10^-6^ M EDTA, 1×10^-5^ M DTPA, 1×10^-5^ M TTHA, and 1×10^-3^ M NaCN) The titration curves of Hg(II) to MerR nearlly have none changes in the presence of competitors compared to the curve generated in the absence of competitors.

**Table S1**. Bond length, bond angle and single point (SP) energy

in *Tn501* MerR and *Bacillus* MerR

|  | | *Tn501* MerR | *Bacillus* MerR |
| --- | --- | --- | --- |
| Hg^2+^-binding site 1 | Bond length | 2.55 Å, 2.57 Å, 2.56 Å | 2.44 Å, 2.44 Å, 2.46 Å |
|  | Bond angle | 116.7°, 126.1°, 117.0° | 116.9°, 111.3°, 130.7° |
|  | SP energy (a.u.) | -2316.94691 | -2316.93140 |
| Hg^2+^-binding site 2 | Bond length | 2.57 Å, 2.55 Å, 2.57 Å | 2.44 Å, 2.44 Å, 2.46 Å |
|  | Bond angle | 114.9°, 127.0°, 118.0° | 110.3°, 124.8°, 124.8° |
|  | SP energy (a.u.) | -2316.94780 | -2316.94218 |

**Materials and Methods**

***Competitive UV-titration of Hg(II) to MerR***

The purified MerR, HgCl_2_, and small molecular Hg(II) ligands EDTA, DTPA, TTHA, and NaCN were prepared in 20 mM sodium citrate, pH 6.0, 20 mM ammonium tartrate, 250 mM NaCl, and 0.5 mM TCEP. The concentration of MerR protein was determined based on UV spectra with the ε_280_=4080 M^-1^ cm^-1^. In each parallel titration experiments, a fixed concentration of the MerR protein was mixed with a certain competitive small molecular Hg(II) ligand. The mixtures of MerR protein and small molecular Hg(II) ligands were divided into equal volumes and titrated with different concentrations of HgCl_2_ with [consistent](javascript:void(0);) volumes. After adding HgCl_2_, the reaction systems were incubated at room temperature, and the UV spectra were determined until the solution achieved competitive equilibrium. The titration curves of Hg(II) to MerR in the presence of competitors were compared to the curve generated in the absence of competitors.

**References**

1 Caguiat, J. J., Watson, A. L. & Summers, A. O. Cd(II)-responsive and constitutive mutants implicate a novel domain in MerR. *J Bacteriol* **181**, 3462-3471 (1999).

2 Corpet, F. Multiple sequence alignment with hierarchical clustering. *Nucleic acids research* **16**, 10881-10890 (1988).

3 Chang, C. C., Lin, L. Y., Zou, X. W., Huang, C. C. & Chan, N. L. Structural basis of the mercury(II)-mediated conformational switching of the dual-function transcriptional regulator MerR. *Nucleic Acids Res* **43**, 7612-7623, doi:10.1093/nar/gkv681 (2015).

4 Changela, A. *et al.* Molecular basis of metal-ion selectivity and zeptomolar sensitivity by CueR. *Science* **301**, 1383-1387 (2003).
